# On Perception and Consciousness in HPPD: A Systematic Review

**DOI:** 10.3389/fnins.2021.675768

**Published:** 2021-08-11

**Authors:** Pieter J. Vis, Anneke E. Goudriaan, Bastiaan C. ter Meulen, Jan Dirk Blom

**Affiliations:** ^1^Department of Neurology, Zaans Medical Centre, Zaandam, Netherlands; ^2^Department of Psychiatry, Amsterdam University Medical Center, Amsterdam, Netherlands; ^3^Outpatient Clinic for Uncommon Psychiatric Syndromes, Parnassia Psychiatric Institute, The Hague, Netherlands; ^4^Amsterdam Institute for Addiction Research, Amsterdam University Medical Center, Amsterdam, Netherlands; ^5^Department of Neurology, Onze Lieve Vrouwe Gasthuis Teaching Hospital, Amsterdam, Netherlands; ^6^Faculty of Social and Behavioural Sciences, Leiden University, Leiden, Netherlands; ^7^Department of Psychiatry, University of Groningen, Groningen, Netherlands

**Keywords:** Alice in Wonderland syndrome, adverse effect, flashback, hallucination, metamorphopsia, perceptual distortion, psychedelic, visual snow

## Abstract

Hallucinogen-persisting perception disorder (HPPD) features as a diagnostic category in the DSM-5, ICD-11, and other major classifications, but our knowledge of the phenomenology of the perceptual symptoms involved and the changes in consciousness during the characteristic “flashbacks” is limited. We systematically evaluated original case reports and case series on HPPD to define its phenomenology, associated (psycho)pathology, and course. Our search of PubMed and Embase yielded 66 relevant publications that described 97 people who, together, experienced 64 unique symptoms of HPPD. Of these, 76% concerned symptoms characteristic of Alice in Wonderland syndrome, over 50% non-visual symptoms, and 38% perceptual symptoms not clearly linked to prior intoxication states. This is in contrast with the DSM-5 diagnostic criteria for HPPD. Even though less than half of the patients showed a protracted disease course of over a year, a third achieved remission. However, in patients with co-occurring depression (with or without anxiety) HPPD symptoms persisted longer and treatment outcomes were more often negative. Thus, unlike the acute stages of psychedelic drug intoxication, which may be accompanied by altered states of consciousness, HPPD is rather characterized by changes in the *content* of consciousness and an attentional shift from exogenous to endogenous phenomena. Since HPPD is a more encompassing nosological entity than suggested in the DSM-5, we recommend expanding its diagnostic criteria. In addition, we make recommendations for clinical practice and future research.

## Introduction

The literature on psychedelic substances almost customarily maintains that these agents produce “altered states of consciousness” (Bayne and Carter, 2018). What is not always clear, though, is whether consciousness is “heightened,” “lowered,” or “narrowed,” or whether the *level* of consciousness is even addressed or, far rather, its content. This is even less clear in states that arise long after the acute effects of the substance use have worn off, which may be days, months, or even years on. In the present paper we seek to understand what happens to consciousness in hallucinogen-persisting perception disorder (HPPD), a condition characterized by perceptual distortions, hallucinations, and other experiential phenomena reminiscent of prior substance use. According to the DSM-5, typical symptoms of HPPD are geometric hallucinations, false perceptions of movement in the peripheral visual fields, flashes of color, intensified colors, trails of images of moving objects, the perception of entire hallucinated objects, positive afterimages, halos around objects, macropsia, and micropsia (American Psychiatric Association, 2013). These manifestations often occur when people look at blank surfaces or move from a well-illuminated space into a darker area. They are primarily associated with the prior use of lysergic acid diethylamide (LSD), but other known triggers include alcohol, cannabis, ketamine, 3,4-methylenedioxymethamphetamine (MDMA), psilocybin, and synthetic cannabinoids (Abraham, 1983; Litjens et al., 2014; Lerner et al., 2014a; Orsolini et al., 2017; Halpern et al., 2018). It is believed that, after a latency period, the onset of symptoms can be triggered by stress and/or renewed substance use, including the use of alcohol (Abraham, 1983; Martinotti et al., 2018).

Of the various health issues caused by the steady, worldwide increase in illicit drug use, HPPD is an underreported and still poorly understood condition (UN Office on Drugs Crime, 2019). Sound prevalence rates are lacking, but the DSM-5 suggests that 4.2% of all hallucinogen users experience HPPD-like symptoms (American Psychiatric Association, 2013). In their literature study, Halpern and Pope (2003) estimate that such symptoms emerge in <5% of all patients treated with LSD-assisted psychotherapy but in up to 50% of polydrug users. Sometimes two subtypes of HPPD are distinguished based on their severity and comorbidity. Type 1 is considered to be the milder variant, where perceptual symptoms are infrequent and barely affect general functioning, with the experiences being predominantly denoted as pleasant (and occasionally as “free trips”). The prognosis is said to be good, with the course often being self-limiting and not requiring professional help (World Health Organization, 2018). Type-2 HPPD, however, is described as causing significant impairment in daily and occupational functioning, while the prognosis is poor, with symptoms lasting up to years or even decades, albeit that large-scale follow-up studies to back this up are scarce (Noushad et al., 2015).

Although it is unknown what proportion of those experiencing HPPD seek professional help, only a small group manages to procure the help they need. This is at least partly due to a lack of knowledge of HPPD among general practitioners and medical specialists. It is widely believed that pharmacological treatment regimens and psychotherapy have little to no effect on HPPD (Lerner et al., 2014c). Since evidence-based treatment guidelines are still to be developed, patients often receive practice-based interventions with off-label medications such as adrenergic agonists, antidepressants, antiepileptics, antipsychotics, benzodiazepines, beta blockers, calcium-channel blockers, catechol-o-methyl transferase inhibitors, and opioid receptor antagonists. The evidence for the effects of these treatments is lacking since they have only been described in case reports and open-label treatment studies.

Studies of HPPD tend to focus on the condition's visual symptoms, but their exact nature has as yet not been systematically reviewed. Notably, also systematic studies of the symptoms characteristic of Alice in Wonderland Syndrome (AIWS) in HPPD are lacking (Blom, 2016, 2020). AIWS is a collective term for visual distortions (also known as metamorphopsias, e.g., macropsia and micropsia), time distortions (e.g., quick- and slow-motion phenomena), and bodily distortions (e.g., macrosomatognosia and microsomatognosia). This “third group of perceptual disorders” (hallucinations and illusions constituting the first two groups) has numerous etiologies, including substance use and a variety of neurological disorders, particularly epilepsy and migraine, and, to a lesser extent, psychiatric disorders (Blom, 2016). We were able to retrieve only one case report in which long-term visual symptoms of LSD use were explicitly identified as being characteristic of AIWS (Lerner and Lev-Ran, 2015). Suspecting that many more of such cases exist, with the present systematic review we aim to further delineate the perceptual phenomenology of HPPD and to shed light on the relationship between the use of specific types of drugs and the nature, severity, and course of HPPD symptoms, the associated (psycho-)pathology, and, finally, to discuss how HPPD symptoms fit in with current paradigms of “altered consciousness.”

## Methods

### Literature Search

For the purpose of the present review we carried out a systematic literature search in PubMed and Embase up until January 1, 2021, using the search terms “hallucinogen-persisting perception disorder,” “post-hallucinogen perception disorder,” “HPPD,” and related terms such as “chronic visual disturbance,” “flashback,” and “recurring hallucination.” We then combined these terms with the names of various hallucinogens and other illicit substances and supplemented the digital searches by backward searches. We included articles in English, Dutch, German, French, and Spanish without date limits as long as the papers reported on original cases or case series on HPPD. Excluded were papers on perceptual symptoms that occurred following the discontinuation of therapeutics (e.g., antidepressants, antiepileptics, and antipsychotics). An exception was made for ketamine, which is used as a therapeutic as well as a recreational drug. Articles on substance users diagnosed with a schizophrenia spectrum disorder or a substance-induced psychotic disorder were only included when HPPD symptoms could be clearly distinguished from the symptoms characteristic of these disorders. Articles about the methamphetamine flashback and alcoholic hallucinosis were excluded because they are both considered psychotic disorders in their own right.

#### Data Extraction

Data extracted from each paper comprised (i) age, (ii) sex, (iii) psychiatric history and comorbidity, (iv) further relevant medical history, (v) suspected triggers, (vi) frequency and duration of drug use, (vii) lifetime drug use, (viii) latency period until HPPD onset, (ix) visual and other perceptual phenomenology, and (x) HPPD symptom course. The perceptual symptoms that we derived were divided into six categories: (1) visual hallucinations, (2) visual illusions, (3) entoptic phenomena, (4) metamorphopsias, (5) “other visual phenomena,” and (6) “other perceptual phenomena.” When informative, some of these categories were divided into subcategories (e.g., simple, geometric, and complex visual hallucinations). Of note, since the original articles did not always differentiate between anxiety and depression as either symptoms or disorders, we from hereon speak of anxiety/anxiety disorder and depression/depressive disorder.

### Qualitative Assessment

To assess the methodological quality of the collected cases and to minimize the risk of bias and the effects of low-quality studies, we used the questionnaire for the Methodological Assessment of the Quality of Case Studies on HPPD (MAQ-HPPD), an instrument we devised for this specific purpose based on the work by Murad et al. (2018) (Supplementary Textbox 1). With the aid of the MAQ-HPPD we verified whether the exposure to the substances and other drugs involved was adequately reported, whether the medical history was complete, and whether there were adequate descriptions of comorbid psychiatric symptoms, latency periods until HPPD onset, and HPPD course and outcome. We also used it to evaluate whether the authors presented sufficient evidence to rule out any alternative explanations accounting for the mediation of the perceptual symptoms they described, such as neurological or psychiatric disorders. With the weighted scoring system of the MAQ-HPPD, publications could attain a maximum quality score of 12.

## Results

### Search Results

**Figure 1:**
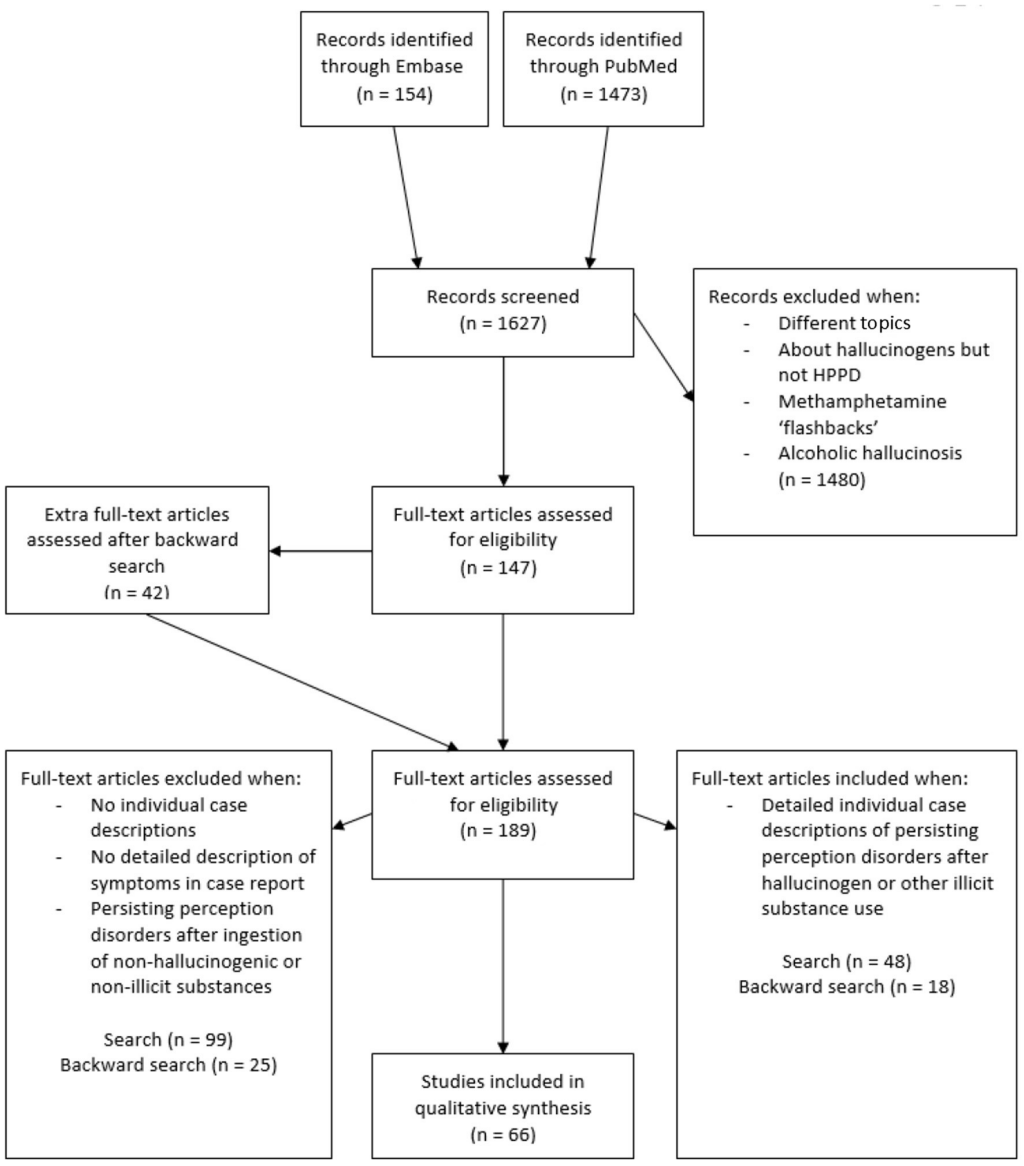
Flowchart of literature search.

Our initial search in Embase and PubMed yielded 1,627 articles of potential relevance. Screening of titles and abstracts led to the exclusion of 1,480 articles that were not related to adverse reactions to substance use. The remaining 147 articles were read in full, after which 99 were excluded, many of which described larger data sets from which individual case descriptions could not be extracted. Through backward searches, an additional 42 articles were identified of which we included 18 case reports. The procedure thus yielded a total of 66 case studies and case series, together describing 97 individual patients (see Figure 1 for flowchart).

### MAQ-HPPD Results

With a mean MAQ-HPPD score of 7.7 out of 12 (median score 7), the methodological quality of 48.4% of the studies was “high” (Table 1). Of the studies not ranked as “high,” the majority scored low on the ascertainment of causality in that they did not provide adequate information in terms of psychiatric and general medical history, psychiatric assessment, neurological/general physical examinations, and/or additional diagnostic details. The suspected causal substance was reported most faithfully, with 82 of the 97 cases (84.5%) getting the maximum score of 12. However, the history of the substance use was incomplete or unclear in 17.5 and 27.8%, respectively. The qualitative assessment of the individual cases can be found in Supplementary Table 1.

**Table 1:** Methodological quality of the case reports as assessed with the MAQ-HPPD.

	**Low quality (0– <4.5)**	**Intermediate quality (≥4.5– <8)**	**High quality (≥8–12)**
No. of individual case reports (%)	6 (6.2%)	44 (45.3%)	47 (48.4%)

### Patient Characteristics

Of the 97 patients reported on, 73% were male and 25% female. In one case, the sex was not indicated. Ages varied from 10 to 49 years, with a mean of 24 years. In 14.4% of the cases, patients were under the age of 18 years. Of the 48 cases (49.5%) with a fully documented psychiatric history, substance-use disorder was the most frequently mentioned condition associated with the onset of HPPD (33.3%). Less common comorbid disorders were depression/depressive disorder (8.3%), anxiety/anxiety disorder (8.3%), psychotic disorder (6.3%), and personality disorder (2.3%). For 44.2% of the patients the psychiatric history had no relevance to the (onset of the) HPPD. The patient's general medical history was documented in 49.5%, of which 4.2% mentioned a history of migraine and 2.1% occipital-lobe epilepsy.

### HPPD Triggers

The substances that were identified most frequently as triggers of HPPD were LSD (37.1%), cannabis (13.4%), and MDMA (6.2%). Other substances, together accounting for 13.4% of the cases, were 4-bromo-2,5-dimethoxyphenethylamine (2C-B), 4-ethyl-2,5-dimethoxyphenethylamine (2C-E), 2,5-dimethoxy-4-methylamphetamine (DOM), ayahuasca, ibogaine, ketamine, mescaline, psilocybin, and synthetic cannabinoids. In 6.2% of the cases HPPD developed after the use of combinations of two or more substances but, since it was not reported whether the symptoms were indicative of a specific substance, it was difficult to pinpoint the likely trigger. Lastly, in 23.7% of the patients the trigger was unknown mostly because there were multiple possibilities.

### Phenomenology of the HPPD Symptoms Reported on

The 97 individual case reports described 64 unique manifestations of HPPD, which are detailed in Table 2, where they are arranged in the six overarching categories we created. Metamorphopsias were the most frequent (67%), followed by “other perceptual phenomena” (52.6%), visual hallucinations (46.4%), visual illusions (26.8%), “other visual phenomena” (18.6%), and entoptic phenomena (including visual snow; 8.4%). In the category “other perceptual symptoms,” only 5.2% of the cases were without accompanying visual phenomena. Table 3 shows the number of symptom categories triggered by the different groups of substances. The hallucinogen group comprised 2-CB, 2-CE, DOM, ibogaine, ketamine, LSD, ayahuasca, mescaline, and psilocybin. LSD use accounted for 78.3% in this category. The group of cannabinoids consisted of cannabis (81.3%) and synthetic cannabinoids (18.7%). MDMA was the only drug in the empathogen group. The 23 patients without a clear trigger were excluded from this analysis. Of the patients who had experienced symptoms in more than three different perceptual categories, the majority had used hallucinogens or combinations of drugs (all of which also included at least one hallucinogen). In 61.9% of the cases the perceptual symptoms were similar to those experienced during previous acute-state trips, while the remaining 38.1% were new perceptual phenomena (i.e., not reminiscent of previous trips). Alterations in consciousness (i.e., heightened, lowered, or narrowed states) were not reported.

**Table 2:** Overview of HPPD symptoms reported in case descriptions (*N* = 97).

**Perceptual disorders**	**Description**	**No. (%) of cases (*N* = 97)**	**Examples from case reports**
1. Visual hallucinations	Visual perception of objects/scenes without an external stimulus	45 (46.4%)	
Simple hallucinations	Hallucinations with the lowest degree of complexity	24 (24.7%)	
Coloropsia	Flashes of color	6 (6.2%)	–
Blank hallucination	Hazy blurrings of perception and cloud-like phenomena	1 (1%)	“*Everything becomes misty, as though I am looking through a fog.”*
Photopsia	Flashes of light	9 (9.3%)	–
Visual snow	Television-like static	14 (14.4%)	“*Everything I saw was covered by grainy colourful dots.”*
Geometric hallucinations	Perception of geometric patterns such as spirals, funnels, tunnels, lattices, cobwebs, or fractals	13 (13.4%)	“*She experienced pleasant shifting panels of light visualized three or four feet in front of her eyes, and the breaking of the light into droplets of color.”*
Closed-eye geometric hallucinations	Exclusively experienced with eyes closed	3 (3.1%)	“*I saw round objects with my eyes closed.”*
Complex hallucinations	Hallucinations with the highest degree of complexity	9 (9.3%)	–
Zoopsia	Hallucinations of animals	4 (4.1%)	“*There was a dark scorpion on the back of my hand that sometimes changed position.”*
Facial hallucinations	Hallucinations of faces	3 (3.1%)	“*There were skulls of familiar faces moving on the walls.”*
Other/unspecified	–	5 (5.2%)	–
Other	–	4 (4.1%)	–
Unspecified hallucinations	–	3 (3.1%)	–
Unspecified closed-eye hallucinations	–	1 (1%)	–
2. Visual illusions	Misperceiving or misinterpreting of visually perceived objects/scenes	26 (26.8%)	
Physiological illusions	Illusions following excessive stimulation of the brain or retina	16 (16.5%)	–
Prolonged negative afterimages	Afterimages in colors complementary to the primary stimulus	15 (15.5%)	–
Other	–	2 (2.1%)	“*Flickering sensation when looking at patterned objects.”*
Cognitive illusions	Illusions following conscious or unconscious assumptions regarding the external environment	6 (6.2%)	–
Pareidolia	Gestalt change	3 (3.1%)	“*He reported seeing faces when staring at trees.”*
Other	–	3 (3.1%)	“*He would see the walls around him tend to crumble. The buildings around began to rise into the air and to swallow up the ground underneath.”*
3. Entoptic phenomena	Visual phenomena caused by ophthalmological pathology	8 (8.4%)	
Mouches volantes	Floaters	7 (7.2%)	–
Blue field entoptic phenomena	Perception of white blood cells projected on the retina, especially against a bright blue background	1 (1%)	“*She described scintillating white dots when looking at a white wall or a blue sky.”*
4. Metamorphopsias	Distortions of visual environmental stimuli	65 (67%)	
Akinetopsia	Inability to perceive motion	1 (1%)	“*Moving objects seemed to have disjointed movements.”*
Chromatopsia	Seeing the external environment in an abnormal hue	4 (4.1%)	“*My vision was like looking at a poorly tuned television with abnormal colors.”*
Corona phenomenon	Perception of an extra contour around objects	15 (15.5%)	–
Dysmetropsia	Change in the apparent size and distance of objects	3 (3.1%)	–
Dysmorphopsia	Lines and contours of objects appear to be wavy	6 (6.2%)	“*Items waved, as though made of jelly.”*
Enhanced stereoscopic vision	Exaggeration of the depth and detail of objects	5 (5.2%)	“*Specks of dust on the ground seemed abnormally obvious.”*
Hyperchromatopsia	Intensification of color	5 (5.2%)	“*Colors were described as vivid and unreal.”*
Illusory visual spread	Expansion of visually perceived patterns	1 (1%)	–
Kinetopsia	Illusory sense of movement	21 (21.6%)	“*Stationary objects moved spontaneously in a pendular manner in all directions.”*
Loss of stereoscopic vision	3D objects appearing to be flat	4 (4.1%)	“*I experienced loss of depth perception.”*
Macropsia	Seeing objects larger	5 (5.2%)	“*His hands seemed larger than they actually were.”*
Micropsia	Seeing objects smaller	4 (4.1%)	“*His dog's head was seen smaller.”*
Oscillopsia	Objects in the visual field appear to oscillate	4 (4.1%)	“*The environment surrounding his point of fixation seemed to move from side to side.”*
Palinopsia	Illusory recurrence of visual perception	12 (12.4%)	“*After looking at any object, a clear and life-like image would persist for several seconds to several minutes.”*
Pelopsia	Seeing objects closer than they are	3 (3.1%)	“*The walls around him seemed like they were closing in.”*
Prosopometamorphopsia	Distorted perception of faces	2 (2.1%)	“*People's faces seemed to change shape.”*
Teleopsia	Seeing objects further than they are	2 (2.1%)	“*The chairs in the room were perceived as further away.”*
Trailing phenomenon	Perception of stationary images trailing behind moving objects	18 (18.6%)	“*He complained of a stroboscopic vision.”*
Visual recursion	Also known as the “Droste effect,” in this case a woman holding an object with a picture of her holding the same object with the same picture and so on	1 (1%)	–
Zoom vision	Intermittent micropsia and macropsia	5 (5.2%)	“*Everything around him started to pulse and breath.”*
Unspecified visual distortions	–	5 (5.2%)	–
5. Other visual phenomena	Visual experiences other than hallucinations, illusions, entoptic phenomena, and metamorphopsias	18 (18.6%)	
Amaurosis fugax	Sudden, temporary blindness	2 (2.1%)	“*Within seconds a complete blindness of both eyes occurred without any signs of psychogenic blindness and no other neurological or ophthalmological abnormalities.”*
Blurred vision	Unclear or cloudy vision	2 (2.1%)	“*A common phenomenon for him was for words on a page he was reading to blur.”*
Hyperesthesia of the retina	Increased sensitivity of the retina to visual stimuli	7 (7.2%)	“*She reported difficulty seeing after something bright flashed in her eyes.”*
Hypnagogic/hypnopompic hallucinations	Hallucinations that occur while falling asleep or waking up	1 (1%)	“*He couldn't sleep because every time he lied down he imagined seeing puffed-up faces and large clocks pointing their hands at him.”*
Impaired night vision	Difficulty seeing in the dark	1 (1%)	–
Prosopagnosia	Inability to recognize faces	1 (1%)	“*He had returning flashbacks where he was not able to recognize himself in the mirror."*
Spontaneous complex imagery	Bonnet-syndrome-like illusions/hallucinations	5 (5.2%)	“*She reported involuntary illusions of people decomposing on the street in front of her.”*
6. Other perceptual phenomena	Non-visual distortions and perceptual disturbances	51 (52.6%)	
Body schema illusions	Misperception of the size or shape of one's body	9 (9.3%)	–
Total-body macrosomatognosia	Perceiving the whole body as larger	1 (1%)	“*Sometimes he felt like he was taller than normal.”*
Partial macrosomatognosia	Perceiving parts of one's body as larger	2 (2.1%)	“*Her arms seemed three times as long.”*
Partial microsomatognosia	Perceiving parts of one's body as smaller	1 (1%)	“*He had the feeling like his head was shrinking.”*
Phantom illusion	Illusory alteration of the shape and/or orientation of the body	1 (1%)	“*She felt like her body was changing shape.”*
Unspecified body schema illusion	–	4 (4.1%)	–
Depersonalization	Experiencing oneself as unreal	11 (11.3%)	“*He felt like going through life as a robot and the sense of his skin having a second layer.”*
Derealization	Experiencing the world as unreal	20 (20.6%)	“*He also became detached from other people and felt like retreating into a different world.”*
Illusory feeling of levitation	Sensation of floating	6 (6.2%)	“She experienced the sensation of being in an elevator.”
Time distortions	Altered perception of time	6 (6.2%)	–
Quick-motion phenomenon	Acceleration of perceived time	2 (2.1%)	“*Viewed objects would speed up, as if the patient were watching a motion picture at fast speed”*
Slow-motion phenomenon	Deceleration of perceived time	2 (2.1%)	“*When people talked, it seemed to the patient that the movements of their mouths were not synchronized with their voices.”*
Other	Bizarre alterations in the perception of time	2 (2.1%)	“*Time would completely stop, then start again”*
Auditory hallucinations	Hearing things in the absence of an external source	4 (4.1%)	“*My TV began talking to me again.”*
Palinacusis	Repetition of externally mediated sounds	3 (3.1%)	“*Sometimes sounds echoed or continued for a long period in his head.”*
Kinesthetic hallucinations	Hallucinatory or illusory sensation of body movement	2 (2.1%)	“*He had kinesthetic images of himself crashing to the window of his car.”*
Olfactory hallucinations	Smelling things in the absence of an external source	1 (1%)	“*He smelled the scent of his mother, whom he had not seen for years.”*
Pain hallucinations	Pain in the absence of physiological stimulation	1 (1%)	“*He experienced a painful sensation of checkerboards passing through his body.”*
Sleep paralysis	Inability to move while waking up or falling asleep	1 (1%)	“*He had a sudden sinking feeling and paralysis. This was accompanied by a rushing sound in his ears, flushing of his face, and the experience of vivid colors and intensified perception. At times he saw spirals which would wrap tightly around his body like the coils of a snake.”*
Sensory disturbances	Hyper- and hypoaesthesia	5 (5.2%)	“*She had a heightened sense of hearing, taste, touch and a blunted pain threshold.”*
Somatic hallucinations	Bodily hallucinations, experienced as if coming from inside the body	1 (1%)	“*He described sensations going through his body such as tingling, heat or cold.”*
Synesthesia	Hallucinations in one sensory modality triggered by sense perceptions in another sensory modality	2 (2.1%)	“*He experienced round objects with pointy protrusions whenever he spoke.”*
Tactile hallucinations	Bodily hallucinations, experienced as coming from outside	3 (3.1%)	“*He felt like an animal had sunk his claws in his back. For the next five days he still felt this.”*
Vestibular hallucinations	Vertigo in the absence of stimulation of the organs of equilibrium	3 (3.1%)	“*She had a continuous feeling of being on a ship.”*

*Numbers do not add up to 100% because several patients experienced multiple symptoms of HPPD*.

**Table 3:** Triggers of HPPD and number of different symptom categories.

**Implied trigger and number of different symptom categories (%)**	**1 category**	**2 categories**	**3 categories**	**4 categories**	**5 categories**	**6 categories**
Hallucinogens (*n* = 46)	14 (30.4%)	15 (32.6%)	13 (28.3%)	3 (6.5%)	1 (2.2%)	0 (0%)
Cannabinoids (*n* = 16)	6 (37.5%)	6 (37.5%)	1 (6.3%)	2 (12.5%)	1 (6.3%)	0 (0%)
Empathogens (*n* = 6)	3 (50%)	1 (16.7%)	0 (0%)	2 (33.3%)	0 (0%)	0 (0%)
Combinations (*n* = 6)	2 (33.2%)	0 (0%)	2 (33.2%)	0 (0%)	2 (33.2%)	0 (0%)

### Psychiatric Comorbidity

The relationship between HPPD symptom type(s) and psychiatric comorbidity was varied. On average, HPPD symptoms were more complex in the presence of comorbid anxiety/anxiety disorder and/or depression/depressive disorder. In the anxiety group, 47.1% of the patients reported HPPD symptoms that fell within three or more different categories; in the depression group this was 44.4% and in the group with both depression and anxiety 57.1%. In the group diagnosed with a substance-use disorder, it was 66.7%, although a similar proportion of these patients also reported symptoms of anxiety or depression, rendering it unclear whether this high rate of complex symptoms should be attributed to the substance-use disorders or to anxiety/depression. Overall, HPPD was less complex in the group without psychiatric comorbidity, where no patients reported symptoms of more than two different categories. In 22.3% of all cases it was unknown whether there were any psychiatric comorbidities.

### Course

To establish the course of the HPPD symptoms described, we assessed the latency period between the substance use and the onset of the symptoms, their frequency and duration, and the outcome (with and without treatment). The *latency period* was documented in 59.8% of the cases, in which in 63.8% the symptoms started relatively early, i.e., immediately (29.3%), within a day (3.4%), or in less than a week (31%). In 12.1% HPPD onset did not occur until a week and in 24.1% more than a month later, with the longest latency interval lasting over 20 years. With regard to *symptom frequency*, in 30.7% of the patients symptoms were continuous, in 34.1% they recurred daily, and in 36.4% symptoms were intermittent with longer intervals between their occurrence. *Symptom duration* was reported in 75.3% of the cases, with lengths amounting to a month up to a year in 41.1%, a year to 5 years in 30.1%, and over 5 years in 16.4%. Only a small minority of the patients (12.3%) experienced HPPD symptoms for less than a month. *Outcome* was described in 74.2% of the cases. Of the 57 patients (58.8%) who had participated in treatment programmes, 63.2% had a positive outcome, with 29.8% of the total number attaining full and 33.3% partial remission. Of the remaining 36.8%, 17.5% was treated without effect, 12.3% reported a reduction in their symptoms but still noted that the remaining symptoms were debilitating, while 7% reported a worsening of their symptoms. Of the 14 patients (14.4%) who had received no treatment, one had persisting symptoms, while in the other 13 cases the HPPD ran a self-limiting course. The HPPD was likewise self-limiting in the cases without comorbid psychiatry.

A high proportion (83.3%) of patients without psychiatric comorbidity showed intermittent HPPD symptoms. This suggests that episodic symptoms and the absence of concurrent psychiatric symptoms are both favorable prognostic factors. Another evident observation we can make is that patients with depression/depressive disorder or anxiety/anxiety disorder, and those with (symptoms of) both conditions, tend to have a longer duration of HPPD and a poorer outcome than those with comorbid anxiety only or those with a formal diagnosis of substance use disorder. Finally, as to the distinction of type-1 and type-2 HPPD, our analysis did not yield any indications on which to found sound prognostic criteria in terms of current symptomatology or associated pathology. At best, the distinction could serve a retrospective purpose in showing that some patients experience a course that significantly impacts their functioning (type 2), whereas others do not (type 1).

## Discussion

In our review of the literature on HPPD, we analyzed 97 detailed case reports that fulfilled our quality criteria. Most conspicuously, none of these cases were characterized by the so-called “altered states of consciousness” that are almost routinely mentioned in the literature on psychedelics. Instead, we found multiple changes pertaining to the *content* of consciousness. The 64 unique perceptual symptoms we identified showed an overlap of 76% with those characteristic of Alice in Wonderland syndrome (AIWS). Before we go on to discuss the implications of this finding for the classification, diagnosis, and treatment of HPPD, we will briefly address some historical issues, and pause to reflect on potential ramifications of our findings for pathophysiology. Finally, we will reprise the issue of consciousness in the context of HPPD.

### Brief Historical Perspective

The history of the use of psychedelics is probably as long as the history of humanity. On the basis of carbon dating of peyote buttons and red beans containing mescaline, found in Mexican caves, it has been suggested that these substances were already used some 5,700 years ago (Bruhn et al., 2002). It is unsure, however, whether knowledge about the long-term effects of these substances is also that old. Due to our limited access to the experiences of ancient cultures where the use of psychedelics (or rather, entheogens) flourished, “flashbacks” and “endless trips” have, as far as we can tell, only been described since the advent of LSD-assisted psychotherapy in the 1950s and the wave of recreational use of psychedelics that ensued during the 1960s (Halpern and Pope, 2003; Orsolini et al., 2017). Of note, the conceptualization of “flashbacks” and “endless trips” is intimately tied up with modern scientific notions about biochemistry and neurophysiology. In ancient cultures where entheogens were rather conceptualized as substances that facilitate “naturally occurring” transcendental states, the notion of a recurring effect of said substances may have been meaningless, and instead have been interpreted as a “naturally occurring transcendental state.” It is only within the boundaries of the scientific framework that terms such as “flashbacks” and “endless trip” have the meaning we now give them. Nonetheless, before these terms had been introduced, Guttman (1924) had already described a mescaline hallucination that he had re-experienced 2 years after the initial effect. And yet it was a report published 30 years later by the British psychiatrist Ronald A. Sandison (1916–2010)—who treated his patients with LSD obtained directly from its inventor, the Swiss chemist Albert Hoffmann (1906–2008)—that drew wider attention to the topic (Sandison et al., 1954). Almost a decade on, more substantial evidence for the long-term effects of hallucinogens became available when Cohen and Ditman (1963) published the results of a survey among 62 scientific experts on mescaline and LSD, 44 of whom provided data on 5,000 people using these substances. An analysis of the data thus obtained indicated that the long-term effects hinted at by authors such as Guttman and Sandison were no fluke. As recounted by Cohen,

*During the days immediately following the drug exposure, certain undesirable effects were seen. One was a prolongation of the hallucinogenic state for twenty-four to forty-eight hours beyond the usual time of termination. These were psychoticlike episodes with agitation and visual aberrations which subsided with psychiatric assistance or tranquilizing medication. Another possibility, especially when mescaline was used, was the transient recurrence of the reaction days or months afterward. Some of these recrudescences were momentary and pleasant, others lasted for minutes or hours and were often construed as distressing* (Cohen, 1964, p. 210).

On the basis of their survey, Cohen and Ditman (1963) gained the impression that such “*prolonged psychotic states occurred in one out of every 550 patients*.” For the American Psychiatric Association the relatively few studies that followed during the next two decades were sufficient to incorporate this group of peculiar perceptual phenomena into the DSM-III-R as “post-hallucinogen perception disorder” (American Psychiatric Association, 1987). From the DSM-IV-TR onwards, the condition is named “hallucinogen-persisting perception disorder” (American Psychiatric Association, 2000).

### Pathophysiology

Although its main focus is on changes in perception and consciousness in HPPD, the current study may also have several implications for our ideas on pathophysiology. As yet, the pathophysiology of HPPD remains to be fully elucidated. That said, there is little doubt that it depends on central rather than peripheral mechanisms. Although studies in the field of ophthalmology indicate that visual distortions such as dysmorphopsia (where lines and contours of objects are perceived as wavy) may also have peripheral causes, it is unlikely that HPPD originates from the retina. Evidence for this stems from the fact that, among other characteristics, geometric hallucinations do not tend to move along with eye movements, while trailing phenomena are limited to one or several objects in the visual field rather than that they affect the visual field as a whole. Moreover, EEG patterns in acute intoxication states—as well as in HPPD patients not under the influence of substances—show faster alpha frequencies and shorter visually-evoked response times, both indicative of cortical disinhibition and thus of neurophysiological changes in the central nervous system (CNS; Abraham and Duffy, 1996, 2001).

These changes may well be partly or fully due to serotonergic effects. With hallucinogens partially agonizing 5-HT2A receptors throughout the brain, chronic cortical disinhibition resulting from damage to inhibitory interneurons is believed to play an important role in the mediation of HPPD (Abraham and Aldridge, 1993). Specifically, the 5HT2 hypothesis suggests the involvement of chronic cortical disinhibition and a dysfunctioning of visual tracts in the thalamus through the destruction of serotonergic inhibitory neurons (Martinotti et al., 2018). This hypothesis is mainly based on the working mechanisms of LSD and other hallucinogens, which achieve their effects through the activation of the 5-HT2A receptor. Likewise, MDMA has an affinity for the 5-HT2A receptor but mainly as a potent releaser and reuptake inhibitor of serotonin. However, this central role of the 5-HT2 hypothesis is challenged by the fact that HPPD can also be triggered by substances with very different working mechanisms. Cannabis, for example, influences the visual pathways by activating the CB1 (cannabinoid) receptor (Litjens et al., 2014), while in AIWS multiple working mechanisms have been suggested. In the latter syndrome, visual and bodily distortions are attributed to mechanisms that act on isolated cortical columns and larger neuron populations with highly specific functions in the detection and representation of perceptual input (e.g., coding for straight lines, distance, size, color, and movement; Blom, 2020). It would seem that these neuron populations are largely under the control of acetylcholine. Time distortions, in turn, would seem to be mainly associated with dopaminergic dysfunction (Rammsayer, 2009; Jones and Jahanshahi, 2011; Blom et al., 2021). How cannabinoids, empathogens, dissociatives, hallucinogens, and stimulants all trigger HPPD symptoms through all those different working mechanisms is, as yet, unknown. Although these issues are all in need of further study, for now we consider it more likely that multiple receptor systems are involved in the pathophysiology of HPPD rather than the serotonergic system alone.

### Implications for Classification

The inclusion of HPPD as a diagnostic category in major classifications such as the DSM-5 is important for clinical practice, research, and the general awareness of the condition among health professionals and lay persons. However, our analysis demonstrates that these classifications wield operational criteria that fail to do justice to the broad symptomatology of HPPD. For example, the A criterion of HPPD in the DSM-5 lists as possible symptoms “*geometric hallucinations, false perceptions of movement in the peripheral visual fields, flashes of color, intensified colors, trails of images of moving objects, positive afterimages, halos around objects, macropsia and micropsia”* (American Psychiatric Association, 2013). This is a mere fraction of the 64 symptoms that we found in our analysis. Historically, the DSM's rather limited inventory of visual disturbances was adapted from a study by Abraham (1983), which reported on the visual symptomatology of “flashbacks” in 125 patients with a history of mainly LSD and cannabis use. Other symptoms and comorbid psychiatric disorders were not considered and, to date, have not been incorporated in the DSM criteria for HPPD even though studies published between 1955 and 1983 already mention the frequent occurrence of perceptual symptoms such as depersonalization, auditory hallucinations, body schema illusions, time distortions, tactile phenomena, spontaneous imagery, and on psychiatric symptoms/disorders in the areas of anxiety and depression (Halpern and Pope, 2003). Later studies described even more symptoms that are peculiarly left unmentioned in the DSM-5, such as acquired dyslexia, closed-eye hallucinations (comprising images and geometric patterns), derealization, distorted perception of distance, ghosted texts, achromatopsia, mouches volantes, negative afterimages, object fragmentation, pareidolia, pattern repetitions, enhanced sharpness of color contrasts, superimposition of geometric patterns, synesthesias, visual snow, and the visualization of dots or particles (Lerner et al., 2014c; Martinotti et al., 2018). In all, our evaluation indicates that classifications of the psychopathology of HPPD might benefit from including a substantially larger number of potential symptoms and associated disorders to raise or expand the awareness of this broad range of symptoms and thus serve both clinical and scientific practice.

Another finding from our study with possible ramifications for the current classifications is that most people with HPPD characterize their symptoms as reminiscent of prior acute trips but that a substantial proportion of 38.1% (also) report new perceptual phenomena, that is, symptoms they never experienced before. Like Lerner et al. (2014b) and Halpern et al. (2018), we conclude that this is in contrast with the DSM-5 criterion which states that HPPD involves the “*reexperiencing [of] one or more of the perceptual symptoms that were experienced while intoxicated with the hallucinogen”* (American Psychiatric Association, 2013). Although this might imply that a clause should be added to the operational definition of HPPD to the effect that symptoms do not always need to be reperceptions of phenomena already experienced before, our finding invites the question of whether such new phenomena should really be attributed to prior substance use, or, alternatively, to an undiagnosed comorbid disorder. Future research is needed to sort this out.

### Clinical Practice

#### Diagnosis

In clinical practice, establishing a diagnosis of HPPD hinges on proper history-taking, which in turn relies on an adequate insight into the condition's wide array of symptoms. Obviously, if substance-use problems are present, substance-use counseling is necessary. In addition, a psychiatric and neurological examination are indispensable, as well as blood work (including toxicology) and a medication review. Only on indication do we recommend an EEG, a head MRI, a lumbar puncture, and/or a neuropsychological assessment.

#### Differential Diagnosis

HPPD is diagnosed when the symptoms are not caused by neurological conditions (e.g., occipital epilepsy, anatomical lesions, or encephalitides of the occipital lobes) and not attributable to another psychiatric disorder (e.g., delirium, dementia, or schizophrenia) or hypnagogia (hallucinations between wakefulness and sleep) (American Psychiatric Association, 2013). Considering the substantial overlap with AIWS, this is yet another condition to be taken into account in the differential diagnosis (Blom, 2016).

#### Treatment

Our finding that 63% of the patients who received pharmacotherapy had a positive outcome—with 30% achieving full remission—may be considered an antidote against therapeutic nihilism in the face of HPPD. Still, we need to realize that these results were obtained with off-label medications and that the absence of evidence-based treatment protocols prevents us from giving any specific advice about which (type of) treatment would best suit individual requirements. One might therefore argue that the therapeutic success of medications in HPPD then depends on clinical expertise. Since our analysis indicates that comorbid psychiatric symptoms and disorders (such as anxiety/anxiety disorder and depression/depressive disorder) negatively influence the course of HPPD, an even more important recommendation may be to treat such comorbid disorders when present. Since patients with comorbid anxiety/anxiety disorder showed better treatment outcomes and shorter symptom durations than those presenting with depression/depressive disorder alone or with both, we feel especially depressive disorder is in need of proper treatment. Although we found little prognostic value in the old type-I/type-II distinction, perhaps the presence or absence of comorbid depression (with or without anxiety) comes closest to what we may currently regard as an indicator of prognosis in HPPD. Lastly, awareness campaigns on the risks of HPPD following substance use may contribute to the prevention of the disorder.

### HPPD and Consciousness

As alluded to above, acute intoxication with psychedelics may be accompanied by a lowered, heightened, or narrowed state of consciousness but our analysis yielded no indications for such changes in HPPD. That said, the experiential phenomena characteristic of this condition do tend to redirect the patient's attention from the content of ordinary sense perception toward the perceptual disturbances involved. Since the content of these disturbances is (more often than not) based on prior sensations experienced during intoxication states, the mechanism involved would seem to comply with the classic reperception model as originally proposed by Kahlbaum (1866). In terms of neural correlates, this would imply involvement of the memory system, notably visual memory. The observation that the symptoms of HPPD are often triggered by looking at blank surfaces or during transitions from well-illuminated to darker spaces suggests that the underlying process also shares characteristics with the dual-input model proposed by West (1962). To explain the model, West used the metaphor of a man looking through a window facing a fireplace, seeing the sun go down and the garden becoming darker, and at the same time observing the reflections emanating from the fireplace getting brighter and brighter. Likewise, HPPD patients apparently become aware of their perceptual symptoms when their regular sensory input diminishes. In his model, West emphasized the role of arousal in the release of such endogenous percepts, saying that “*The greater the level of arousal, the more vivid the hallucinations”* (West, 1962). Applied to the phenomenology of HPPD, we propose that HPPD is characterized by changes in the content of consciousness rather than consciousness itself, which is probably subserved by brain processes that heighten or at least redirect one's attention, more specifically, fronto-parietal networks that provide focal awareness of specific types of perceptual input, whether it be exogenous or endogenous (Nani et al., 2019). HPPD symptoms such as derealization and depersonalization might be exceptions to this tenet because of the narrowing of consciousness that characterizes these phenomena.

### Future Research

In the light of our findings, we would like to see future research attempt to solve various theoretical and practical issues. We first need to establish the incidence and prevalence of HPPD in the general population and populations at risk. To facilitate a proper understanding of the outcomes of such surveys, we need to formulate and use a rigorous and well-operationalized classification of the perceptual phenomena of HPPD. To this end, we advocate the development of a standardized questionnaire as well as an adjustment of the diagnostic criteria of HPPD in major classifications and textbooks. Preferably, the DSM-5's A Criterion of HPPD should be expanded to include the other distinctive perceptual symptoms we retrieved from the literature and a new criterion added to underscore the impact of comorbid psychiatric symptoms and disorders, most notably anxiety and depressive disorders. The desirability of a criterion pointing to the possibility that perceptual symptoms of HPPD are not always reperceptions of sensations experienced during prior intoxication states deserves further study. Regarding basic research, a further elucidation of the (likely multiple) mechanisms of action underlying HPPD is much needed, as well as an explanation of the latency period until symptom onset and the risk factors involved. We consider research into the role of attention a priority in this context. We, moreover, advocate to promote awareness of HPPD and its numerous manifestations among health professionals, psychedelics users, and those who care for them, so as to prevent unnecessary delays in the diagnosis and—if necessary or desirable—practice-based treatment. Even though the relatively high rate of positive treatment outcomes we found may perhaps be a spurious finding, we do recommend systematic studies of pharmacological and non-pharmacological interventions, with the ultimate aim of developing evidence-based treatment guidelines. Finally, future research should focus on the prognostic implications of co-morbid psychiatric symptoms and disorders (and the effects of treating these first or concurrently) on the development and course of HPPD. Findings may even help to augment the historical distinction of type I vs. type II HPPD, which would, rather than being based on a mere retrospective assessment of outcomes, be formulated in diagnostic terms.

### Limitations

The present review relied on published case reports and case series, the quality of which in large part determined the quality of our analysis. Although almost half of the papers were deemed of high methodological quality, this still is a factor that needs to be taken into account. Also, because none of the case reports used a standardized HPPD interview, the risk of recall bias cannot be eliminated, which risk is already high since patients often needed to describe experiences that happened months or years ago. In terms of outcome, publication bias must have played a role. After all, the likelihood that successful treatments are reported on by individual authors—and subsequently published in scientific journals—is probably much larger than it is for failed treatments. Finally, the observed association between prognosis and anxiety and/or depressive symptoms or disorders does not inform us about the direction of causality. To answer that question, longitudinal studies are necessary.

### Conclusions

Our systematic review of 97 case descriptions of HPPD published to date shows that this late-onset complication of hallucinogen use is characterized by 64 unique perceptual phenomena, most of which are perceptual distortions. In that sense, HPPD shows a phenomenological overlap of 76% with Alice in Wonderland syndrome. Other perceptual symptoms, such as hallucinations and illusions, were less prevalent, although non-visual symptoms were present in over half of the cases. In 38% perceptual symptoms were not clearly linked to prior substance use. Given that major classifications such as the DSM-5 and ICD-11 list only a fraction of these symptoms as diagnostic criteria for HPPD while stating that these are invariantly reminiscent of prior trips, we advise to incorporate the presented findings in future editions. We did not find confirmation for the oft-mentioned presumption that HPPD is characterized by “alterations of consciousness,” other than that the *content* of consciousness is (partially and/or temporarily) changed. Neither did we identify prognostic criteria that justify the current distinction between type-I and type-II HPPD. We did find that comorbid anxiety/anxiety disorder and especially depression/depressive disorder (with or without anxiety) are associated with a longer duration of HPPD symptoms and with poorer treatment outcomes. Since we also found that 63% of the patients who received pharmacotherapy benefited from the medication (30% full remission), we recommend practice-based treatment, especially of any comorbid mental disorders. Pathophysiologically, we suggest that multiple neurotransmitter systems—rather than the 5HT2 system alone—are implicated in the extremely diverse symptomatology of HPPD. A final common pathway might well involve an attentional shift from exogenous toward endogenous perceptual content, in conformity with Kahlbaum's classic reperception model and West's dual-input model. Regarding future research, we recommend promoting awareness of HPPD and its full range of manifestations to thus facilitate epidemiological, diagnostic, pathophysiological, and treatment studies, with the ultimate aim of developing evidence-based treatment guidelines. Finally, studies of the prognostic implications of co-morbid psychiatric symptoms and disorders (and the upshot of their treatment) might aid the rephrasing of type-I and type-II HPPD in diagnostic terms rather than in terms of retrospective outcome analyses.

## Data Availability Statement

The original contributions presented in the study are included in the article and Supplementary Material, further inquiries can be directed to the corresponding author.

## Author Contributions

PV and JDB contributed to the conception and design of the work, analysis and interpretation of data for the work, drafted and revised the work, gave approval for the final version to be published, and agreed to be accountable for all aspects of the work in ensuring that questions related to the accuracy or integrity of any part of the work are appropriately investigated and resolved. AG contributed to the design of the work, to the interpretation of the data for the work, revised the work, gave approval for the final version to be published, and agreed to be accountable for all aspects of the work in ensuring that questions related to the accuracy or integrity of any part of the work are appropriately investigated and resolved. BtM contributed to the conception and design of the work, revised the work, gave approval for the final version to be published, and agreed to be accountable for all aspects of the work in ensuring that questions related to the accuracy or integrity of any part of the work are appropriately investigated and resolved. All authors contributed to the article and approved the submitted version.

## Conflict of Interest

The authors declare that the research was conducted in the absence of any commercial or financial relationships that could be construed as a potential conflict of interest.

## Publisher's Note

All claims expressed in this article are solely those of the authors and do not necessarily represent those of their affiliated organizations, or those of the publisher, the editors and the reviewers. Any product that may be evaluated in this article, or claim that may be made by its manufacturer, is not guaranteed or endorsed by the publisher.
