# Supplementary material for: On Perception and Consciousness in HPPD: A Systematic Review
Source: Front Neurosci. 2021 Aug 11;15:675768. doi: 10.3389/fnins.2021.675768 (PMC8385145; doi:10.3389/fnins.2021.675768)
Supplement: Supplementary file 1 [file Table_1.docx]

**Table S1 Quality of case reports on HPPD, as assessed with the MAQ-HPPD (N=97)**

| Study | Case no. | | 1a | 1b | 2a | 2b | 2c | 3a | 3b | 3c | 3d | 3e | Total score |
| --- | --- | --- | --- | --- | --- | --- | --- | --- | --- | --- | --- | --- | --- |
| Abraham & Mamen, 1996 | 1 | | 2 | 0 | 1 | 1 | 1 | 1 | 0 | 1 | 0 | 0 | 7 |
| Abraham & Mamen, 1996 | 2 | | 2 | 0 | 1 | 1 | 1 | 1 | 0 | 1 | 0 | 0 | 7 |
| Abraham & Mamen, 1996 | 3 | | 2 | 0 | 1 | 1 | 1 | 1 | 0 | 1 | 0 | 0 | 7 |
| Alarcon et al., 1982 | 4 | | 2 | 1 | 1 | 1 | 1 | 1 | 1 | 1 | 1 | 1 | 11 |
| Alderliefste, 2016 | 5 | | 2 | 1 | 1 | 0,5 | 0,5 | 0 | 0 | 0 | 0 | 0 | 5 |
| Alderliefste, 2016 | 6 | | 1 | 2 | 1 | 0,5 | 0,5 | 0 | 0 | 0 | 0 | 0 | 5 |
| Alderliefste, 2016 | 7 | | 1 | 2 | 0 | 0,5 | 0,5 | 0 | 0 | 0 | 0 | 0 | 4 |
| Aldurra & Crayton, 2001 | 8 | | 1 | 1 | 1 | 0,5 | 1 | 0 | 0 | 0 | 0 | 0 | 4,5 |
| Anderson & O’Malley, 1972 | 9 | | 1 | 1 | 0 | 0 | 1 | 1 | 0 | 1 | 0 | 0 | 5 |
| Anderson et al., 2018 | 10 | | 2 | 1 | 1 | 1 | 1 | 1 | 1 | 0 | 0 | 1 | 9 |
| Benjamin, 1979 | 11 | | 2 | 1 | 1 | 1 | 1 | 1 | 0 | 0 | 0 | 0 | 7 |
| Bernhard & Ulrich, 2009 | 12 | | 2 | 2 | 1 | 1 | 1 | 0 | 1 | 0 | 1 | 1 | 10 |
| Bialos, 1970 | 13 | | 2 | 0 | 0 | 0 | 0,5 | 0 | 0 | 0 | 0 | 0 | 3,5 |
| Brodrick & Mitchell, 2015 | | 14 | 2 | 1 | 1 | 0,5 | 1 | 1 | 1 | 1 | 0 | 1 | 8,5 |
| Brown & Stickgold, 1976 | 15 | | 2 | 2 | 1 | 1 | 1 | 0 | 0 | 0 | 0 | 0 | 7 |
| Brown & Stickgold, 1976 | 16 | | 2 | 2 | 1 | 1 | 0 | 0 | 0 | 0 | 0 | 0 | 6 |
| Brown & Stickgold, 1976 | 17 | | 2 | 2 | 0 | 1 | 0 | 0 | 0 | 0 | 0 | 0 | 5 |
| Brown & Stickgold, 1976 | 18 | | 1 | 2 | 0 | 1 | 1 | 0 | 0 | 0 | 0 | 0 | 5 |
| Charre et al., 2020 | 19 | | 2 | 2 | 1 | 0 | 1 | 1 | 1 | 1 | 0 | 1 | 9 |
| Cohen, 1966 | 20 | | 2 | 1 | 1 | 1 | 1 | 0 | 0 | 0 | 0 | 0 | 6 |
| Cohen & Ditman, 1963 | 21 | | 2 | 1 | 1 | 0 | 1 | 0 | 0 | 0 | 0 | 0 | 5 |
| Cohen & Ditman, 1963 | 22 | | 2 | 2 | 1 | 1 | 1 | 0 | 0 | 0 | 0 | 0 | 7 |
| Cohen & Ditman, 1963 | 23 | | 2 | 0 | 1 | 1 | 0,5 | 0 | 0 | 0 | 0 | 0 | 4,5 |
| Coppola & Mondola, 2017 | 24 | | 2 | 2 | 1 | 1 | 1 | 1 | 1 | 1 | 0 | 1 | 11 |
| Creighton et al., 1991 | 25 | | 2 | 2 | 1 | 0 | 0,5 | 0 | 0 | 1 | 0 | 0 | 6,5 |
| Creighton et al., 1991 | 26 | | 2 | 1 | 1 | 0 | 0,5 | 0 | 0 | 1 | 0 | 0 | 5,5 |
| Ellison-Wright & Sessa, 2015 | 27 | | 2 | 1 | 1 | 1 | 1 | 1 | 1 | 0 | 1 | 1 | 10 |
| Espiard et al., 2005 | 28 | | 2 | 1 | 1 | 1 | 1 | 1 | 1 | 1 | 0 | 1 | 10 |
| Favazza & Domino, 1969 | 29 | | 2 | 1 | 1 | 1 | 1 | 1 | 0 | 1 | 1 | 1 | 10 |
| Gaillard & Borruat, 2003 | 30 | | 2 | 2 | 0 | 1 | 1 | 0 | 0 | 0 | 1 | 1 | 7,5 |
| Gaillard & Borruat, 2003 | 31 | | 1 | 2 | 0 | 0 | 0,5 | 0 | 0 | 0 | 1 | 1 | 5,5 |
| Gaillard & Borruat, 2003 | 32 | | 2 | 1 | 0 | 1 | 1 | 0 | 0 | 0 | 1 | 1 | 7 |
| Hanck & Schellekens, 2013 | 33 | | 2 | 2 | 1 | 1 | 1 | 1 | 1 | 0 | 0 | 0 | 9 |
| Hasse & Waldmann, 1971 | 34 | | 2 | 1 | 1 | 0 | 0,5 | 0 | 1 | 1 | 0 | 0 | 6,5 |
| Hermle et al., 2012 | 35 | | 2 | 2 | 1 | 1 | 1 | 0 | 0 | 1 | 1 | 1 | 10 |
| Hermle et al., 2015 | 36 | | 2 | 2 | 1 | 1 | 1 | 1 | 0 | 0 | 1 | 1 | 10 |
| Hermle et al., 2015 | 37 | | 2 | 2 | 0 | 1 | 1 | 0 | 0 | 1 | 1 | 0 | 8 |
| Horowitz, 1969 | 38 | | 2 | 1 | 0 | 0 | 0 | 0 | 0 | 0 | 0 | 0 | 3 |
| Horowitz, 1969 | 39 | | 2 | 1 | 0 | 0 | 0 | 0 | 0 | 0 | 0 | 0 | 3 |
| Horowitz, 1969 | 40 | | 2 | 2 | 0 | 1 | 1 | 0 | 0 | 0 | 0 | 0 | 6 |
| Iaria et al., 2010 | 41 | | 2 | 2 | 1 | 0,5 | 1 | 1 | 0 | 1 | 1 | 1 | 10,5 |
| Johnson, 1971 | 42 | | 2 | 0 | 1 | 1 | 0 | 0 | 0 | 1 | 0 | 0 | 6 |
| Kaminer & Hrecznyj, 1991 | 43 | | 2 | 2 | 1 | 0 | 1 | 1 | 1 | 1 | 1 | 1 | 11 |
| Kawasaki & Purvin, 1996 | 44 | | 2 | 0 | 0 | 1 | 1 | 0 | 0 | 0 | 1 | 1 | 6 |
| Kawasaki & Purvin, 1996 | 45 | | 2 | 0 | 0 | 1 | 0 | 1 | 1 | 0 | 1 | 1 | 7 |
| Kawasaki & Purvin, 1996 | 46 | | 2 | 2 | 0 | 0 | 1 | 1 | 1 | 0 | 1 | 1 | 9 |
| Keeler et al., 1968 | 47 | | 2 | 0 | 1 | 1 | 1 | 0 | 0 | 1 | 0 | 0 | 5 |
| Keeler et al., 1968 | 48 | | 2 | 0 | 1 | 1 | 1 | 0 | 0 | 1 | 0 | 0 | 6 |
| Keeler et al., 1968 | 49 | | 2 | 0 | 1 | 1 | 1 | 0 | 0 | 1 | 0 | 0 | 6 |
| Keeler et al., 1968 | 50 | | 2 | 0 | 1 | 1 | 0 | 0 | 0 | 1 | 0 | 0 | 5 |
| Kleber, 1967 | 51 | | 2 | 0 | 1 | 1 | 1 | 0 | 0 | 0 | 0 | 0 | 5 |
| Knuijver et al., 2018 | 52 | | 2 | 2 | 1 | 1 | 1 | 1 | 0 | 1 | 1 | 1 | 11 |
| Kurtom et al., 2019 | 53 | | 2 | 2 | 1 | 1 | 1 | 1 | 1 | 1 | 0 | 0 | 10 |
| Lerner et al., 2014a | 54 | | 2 | 2 | 1 | 1 | 1 | 1 | 1 | 1 | 1 | 1 | 12 |
| Lerner et al., 2014a | 55 | | 2 | 2 | 1 | 1 | 1 | 1 | 1 | 1 | 1 | 1 | 12 |
| Lerner et al., 2014b | 56 | | 2 | 2 | 1 | 1 | 1 | 1 | 1 | 1 | 1 | 1 | 12 |
| Lerner et al., 2014b | 57 | | 2 | 2 | 1 | 1 | 1 | 1 | 1 | 1 | 1 | 1 | 12 |
| Lerner et al., 2015 | 58 | | 2 | 2 | 1 | 1 | 1 | 1 | 1 | 1 | 1 | 1 | 12 |
| Lerner et al., 1998 | 59 | | 1 | 2 | 1 | 0 | 1 | 1 | 1 | 0 | 0 | 0 | 7 |
| Lerner et al., 1997 | 60 | | 1 | 2 | 1 | 0 | 1 | 1 | 1 | 0 | 0 | 0 | 7 |
| Lerner et al., 2002 | 61 | | 1 | 2 | 1 | 0,5 | 1 | 1 | 1 | 1 | 1 | 1 | 10,5 |
| Lerner et al., 2001 | 62 | | 2 | 2 | 1 | 1 | 1 | 1 | 1 | 1 | 0 | 0 | 10 |
| Lerner et al., 2001 | 63 | | 2 | 2 | 1 | 0,5 | 1 | 1 | 1 | 1 | 0 | 0 | 9,5 |
| Levi & Miller, 1990 | 64 | | 2 | 2 | 0 | 0,5 | 0,5 | 0 | 1 | 0 | 1 | 1 | 8 |
| Levi & Miller, 1990 | 65 | | 2 | 2 | 0 | 0,5 | 1 | 0 | 1 | 0 | 1 | 1 | 8,5 |
| Levi & Miller, 1990 | 66 | | 2 | 2 | 1 | 1 | 1 | 0 | 1 | 1 | 1 | 1 | 11 |
| Levi & Miller, 1990 | 67 | | 2 | 2 | 0 | 1 | 1 | 0 | 1 | 0 | 1 | 1 | 9 |
| Levi & Miller, 1990 | 68 | | 2 | 1 | 0 | 1 | 1 | 0 | 1 | 0 | 1 | 1 | 8 |
| Levi & Miller, 1990 | 69 | | 2 | 2 | 0 | 1 | 0,5 | 0 | 1 | 0 | 1 | 1 | 8,5 |
| Markel et al., 1994 | 70 | | 2 | 2 | 1 | 0,5 | 1 | 1 | 1 | 0 | 1 | 0 | 9,5 |
| Metzler & Robertson, 2017 | 71 | | 2 | 2 | 0 | 1 | 0 | 1 | 0 | 0 | 0 | 0 | 6 |
| Morehead, 1997 | 72 | | 2 | 1 | 1 | 0,5 | 1 | 1 | 1 | 1 | 1 | 1 | 10,5 |
| Muquebil et al., 2015 | 73 | | 2 | 2 | 1 | 1 | 1 | 0 | 0 | 0 | 0 | 0 | 7 |
| Neven & Blom, 2014 | 74 | | 2 | 2 | 1 | 0 | 1 | 1 | 1 | 1 | 1 | 1 | 11 |
| Noushad et al., 2015 | 75 | | 2 | 2 | 1 | 1 | 1 | 0 | 1 | 1 | 0 | 1 | 10 |
| Nutting et al., 2020 | 76 | | 2 | 1 | 1 | 1 | 1 | 1 | 0 | 1 | 0 | 1 | 9 |
| Passie et al., 2002 | 77 | | 2 | 2 | 1 | 0 | 1 | 1 | 1 | 1 | 1 | 1 | 11 |
| Rosenthal, 1964 | 79 | | 2 | 2 | 1 | 0,5 | 1 | 0 | 0 | 1 | 0 | 0 | 7,5 |
| Saidel & Babineau, 1976 | 80 | | 2 | 2 | 1 | 0,5 | 1 | 0 | 0 | 0 | 0 | 0 | 6,5 |
| Schatten 2020 | 81 | | 1 | 1 | 1 | 1 | 0,5 | 1 | 1 | 0 | 1 | 1 | 8,5 |
| Scott, 1971 | 82 | | 1 | 0 | 1 | 0,5 | 0 | 0 | 0 | 0 | 0 | 0 | 2,5 |
| Scott, 1971 | 83 | | 1 | 0 | 1 | 1 | 1 | 0 | 0 | 0 | 0 | 0 | 4 |
| Skryabin et al., 2018 | 84 | | 2 | 2 | 1 | 1 | 1 | 0 | 0 | 0 | 0 | 1 | 8 |
| Skryabin et al., 2018 | 85 | | 2 | 2 | 1 | 1 | 1 | 1 | 0 | 0 | 1 | 1 | 10 |
| Skryabin et al., 2018 | 86 | | 2 | 1 | 1 | 1 | 1 | 1 | 0 | 0 | 0 | 1 | 8 |
| Stanciu & Penders, 2016 |  | | 2 | 0 | 1 | 1 | 1 | 1 | 0 | 1 | 0 | 1 | 8 |
| Subramanian & Doran, 2013 | 87 | | 2 | 2 | 1 | 1 | 1 | 1 | 1 | 0 | 0 | 0 | 9 |
| Sullivan, 2013 | 88 | | 2 | 1 | 0 | 1 | 0,5 | 1 | 1 | 0 | 0 | 0 | 6,5 |
| Sunness, 2004 | 89 | | 1 | 1 | 0 | 0,5 | 0 | 1 | 1 | 0 | 0 | 1 | 5,5 |
| Thurlow & Girvin, 1971 | 90 | | 2 | 1 | 1 | 0,5 | 1 | 0 | 1 | 0 | 1 | 1 | 8,5 |
| Wentworth-Rohr, 1970 | 91 | | 2 | 0 | 1 | 1 | 1 | 0 | 0 | 0 | 0 | 0 | 5 |
| Wodarz & Böning, 1993 | 92 | | 2 | 2 | 1 | 1 | 1 | 0 | 0 | 1 | 0 | 1 | 9 |
| Woody, 1970 | 93 | | 1 | 2 | 1 | 0 | 0 | 1 | 0 | 0 | 0 | 0 | 5 |
| Woody, 1970 | 94 | | 1 | 2 | 1 | 0 | 0 | 1 | 0 | 0 | 0 | 0 | 5 |
| Young, 1997 | 95 | | 2 | 1 | 1 | 0,5 | 1 | 1 | 1 | 0 | 0 | 0 | 7,5 |
| Zobor et al., 2015 | 96 | | 2 | 2 | 0 | 0,5 | 0,5 | 1 | 0 | 0 | 1 | 1 | 8 |
| Zweben et al., 1994 | 97 | | 2 | 1 | 1 | 1 | 1 | 0 | 0 | 0 | 0 | 0 | 6 |

**References**

Abraham, H.D., and Mamen, A. (1996). LSD-like panic from risperidone in post-LSD visual disorder. *J. Clin. Psychopharmacol.* 16, 238-241.

Alarcon, R.D., Dickinson, W.A., Dohn, H.H. (1982). Flashback phenomena. Clinical and diagnostic dilemmas. *J. Nerv. Ment. Dis*. 170, 217-223.

Alderliefste, G.J. (2016). DPS en HPPD: Signalering, diagnostiek en behandeling van persistente waarnemingsstoornissen na partydrugs. *Verslaving*. 12, 172-184.

Aldurra, G., and Crayton, J.W. (2001). Improvement of hallucinogen persisting perception disorder by treatment with a combination of fluoxetine and olanzapine: Case report. *J. Clin. Psychopharmacol*. 21, 343-344.

Anderson, L., Lake, H., Walterfang, M. (2018). The trip of a lifetime: Hallucinogen persisting perceptual disorder. *Australas. Psychiatry*. 26, 11-12.

Anderson, W., and O'Malley, J. (1972). Trifluoperazine for the "trailing" phenomenon. *JAMA*. 220, 1244-1245.

Benjamin, C. (1979). Persistent psychiatric symptoms after eating psilocybin mushrooms. *Br. Med. J.* 1, 1319-1320.

Bernhard, M.K., and Ulrich, K. (2009). Rezidivierende kortikale Blindheit nach LSD-Einnahme. *Fortschr. Neurol. Psychiatr*. 77, 102-104.

Bialos, D.S. (1970). Adverse marijuana reactions: A critical examination of the literature with selected case material. *Am. J. Psychiatry*. 127, 819-823.

Brodrick, J., and Mitchell, B.G. (2015). Hallucinogen persisting perception disorder and risk of suicide. *J. Pharm. Pract*. 29, 431-434.

Brown, A., and Stickgold, A. (1976). Marijuana flashback phenomena. *Journal of Psychedelic Drugs*. 8, 275-283.

Charre, M., Herrero, H., Martelli, C., Benyamina, A. (2020). Syndrome post-hallucinogène persistant: Discussion à propos d’une situation clinique. *Encephale*. 46, 408-409.

Cohen, S. (1966). A classification of LSD complications. *Psychosomatics*. 7, 182-186.

Cohen, S., and Ditman, K.S. (1963). Prolonged adverse reactions to lysergic acid diethylamide. *Arch. Gen. Psychiatry*. 8, 475-480.

Coppola, M., and Mondola, R. (2017). JWH-122 Consumption adverse effects: A case of hallucinogen persisting perception disorder five-year follow-up. *J. Psychoactive. Drugs.* 49, 262-265.

Creighton, F.J., Black, D.L., Hyde, C.E. (1991). 'Ecstasy' psychosis and flashbacks. *Br. J. Psychiatry*. 159, 713-715.

Ellison‐Wright, Z., and Sessa, B. (2015). A persisting perception disorder after cannabis use. *Prog. Neurol. Psychiatry*. 19, 10-13.

Espiard, M.-L., Lecardeur, L., Abadie, P., Halbecq, I., Dollfus, S. (2005). Hallucinogen persisting perception disorder after psilocybin consumption: A case study. *Eur. Psychiatry*. 20, 458-460.

Favazza, A.R., and Domino, E.F. (1969). Recurrent LSD experience (flashbacks) triggered by marihuana. *Univ. Mich. Med. Cent. J.* 35, 214-216.

Gaillard, M.C., and Borruat, F.X. (2003). Persistierende visuelle Illusionen und Halluzinationen bei zuvor drogenabhängigen Patienten*.* *Klin. Monbl. Augenheilkd*. 220, 176-178.

Hanck, L., and Schellekens, A.F.A. (2013). Persisterende waarnemingsstoornissen na het gebruik van ecstasy. *Ned. Tijdschr. Geneeskd.* 157, A5649.

Hasse, H.E., and Waldmann, H. (1971). „Flashback”: Spontane psychotische Episoden als Folgeerscheinung des Phantasticagebrauchs Jugendlicher. *Arch. Psychiatr. Nervenkr*. 214, 399-439.

Hermle, L., Ruchsow, M., Täschner, K.L. (2015). Halluzinogen-induzierte peristierende Wahrnehmungsstörung (HPPD) and Flashback-Phänomene – Differenzialdiagnose und Erklärungsmodelle. *Fortschr. Neurol. Psychiatr*. 83, 506-515.

Hermle, L., Simon, M., Ruchsow, M., Geppert, M. (2012). Hallucinogen-persisting perception disorder. *Ther. Adv. Psychopharmacol*. 2, 199-205.

Horowitz, M.J. (1969). Flashbacks: Recurrent intrusive images after the use of LSD. *Am. J. Psychiatry*. 126, 565-569.

Iaria, G., Fox, C.J., Scheel, M., Stowe, R.M., Barton, J.J.S. (2010). A case of persistent visual hallucinations of faces following LSD abuse: A functional Magnetic Resonance Imaging study. *Neurocase*. 16, 106-118.

Johnson, B.D. (1971). Psychosis and ketamine. *Br. Med. J*. 4, 428-429.

Kaminer, Y., and Hrecznyj, B. (1991). Lysergic acid diethylamide-induced chronic visual disturbances in an adolescent. *J. Nerv. Ment. Dis*. 179, 173-174.

Kawasaki, A., and Purvin, V. (1996). Persistent palinopsia following ingestion of lysergic acid diethylamide (LSD). *Arch. Ophthalmol*. 114, 47-50.

Keeler, M.H., Reizler, C.B., Liptzin, M.B. (1968). Spontaneous recurrence of marihuana effect. *Am. J. Psychiatry*. 125, 140-142.

Kleber, H. (1967). Prolonged adverse reactions from unsupervised use of hallucinogenic drugs. *J. Nerv. Ment. Dis*. 144, 308-319.

Knuijver, T., Belgers, M., Markus, W., Verkes, R.-J., van Oosteren, T., Schellekens, A. (2018). Hallucinogen persisting perception disorder after ibogaine treatment for opioid dependence. *J. Clin. Psychopharmacol*. 38, 646-648.

Kurtom, M., Henning, A., Espiridion, E.D. (2019). Hallucinogen-persisting perception disorder in a 21-year-old man. *Cureus*. 11, e4077.

Lerner, A.G., Finkel, B., Oyffe, I., Merenzon, I., Sigal, M. (1998). Clonidine treatment for hallucinogen persisting perception disorder. *Am. J. Psychiatry*. 155, 1460.

Lerner, A.G., Goodman, C., Bor, O., Rudinski, D., Lev-Ran, S. (2014a). Synthetic cannabis substances (SPS) use and hallucinogen persisting percerption disorder (HPPD): Two case reports. *Isr. J. Psychiatry. Relat. Sci*. 51, 277-280.

Lerner, A.G., Goodman, C., Rudinski, D., Lev-Ran, S. (2014b). LSD Flashbacks - The appearance of new visual imagery not experienced during initial intoxication: Two case reports. *Isr. J. Psychiatry. Relat. Sci*. 51, 307-309.

Lerner, A.G., and Lev-Ran, S. (2015). LSD-associated "Alice in Wonderland Syndrome"(AIWS): A hallucinogen persisting perception disorder (HPPD) case report. *Isr. J. Psychiatry. Relat. Sci*. 52, 67-68.

Lerner, A.G., Oyefe, I., Isaacs, G., Sigal, M. (1997). Naltrexone treatment of hallucinogen persisting perception disorder. *Am. J. Psychiatry*. 154, 437.

Lerner, A.G., Shufman, E., Kodesh, A., Rudinski, D., Kretzmer, G., Sigal, M. (2002). Risperidone-associated, benign transient visual disturbances in schizophrenic patients with a past history of LSD abuse. *Isr. J. Psychiatry. Relat. Sci*. 39, 57-60.

Lerner, A.G., Skladman, I., Kodesh, A., Sigal, M., Shufman, E. (2001). LSD-induced hallucinogen persisting perception disorder treated with clonazepam: Two case reports. *Isr. J. Psychiatry. Relat. Sci*. 38, 133-136.

Levi, L., and Miller, N.R. (1990). Visual illusions associated with previous drug abuse. *J. Clin. Neuroopthalmol*. 10, 103-110.

Markel, H., Lee, A., Holmes, R.D., Domino, E.F. (1994). LSD flashback syndrome exacerbated by selective serotonin reuptake inhibitor antidepressants in adolescents. *J. Pediatr*. 125, 817-819.

Metzler, A.I., and Robertson, C.E. (2018). Visual snow syndrome: Proposed criteria, clinical implications, and pathophysiology. *Curr. Neurol. Neurosci. Rep*. 18, 52.

Morehead, D.B. (1997). Exacerbation of hallucinogen-persisting perception disorder with risperidone. *J. Clin. Psychopharmacol*. 17, 327-328.

Muquebil Ali Al Shaban Rodríguez, O.W., Álvarez de Morales Gómez-Moreno, E., López Fernández, J.R. (2015). Trastorno perceptivo persistente por alucinógenos: A propósito de un caso. *Psiquiatría Biológica*. 22, 56-58.

Neven, A., and Blom, J.D. (2014). Synesthesieën in het kader van de persisterende waarnemingsstoornis door hallucinogenen na gebruik van lsd. *Tijdschr. Psychiatr*. 56, 748-752.

Noushad, F., Al Hillawi, Q., Siram, V., Arif, M. (2015). 25 years of hallucinogen persisting perception disorder - A diagnostic challenge*.* *B.J.M.P*. 8, 37-39.

Nutting, S., Bruinsma, T., Anderson, M., Jolly, T. (2020). Psychotic and still tripping—Hallucinogen persisting perception disorder and first break psychosis in an adolescent. *Int. J. Ment. Health. Addict*. Doi:10.1007/s11469-020-00338-5.

Passie, T., Schneider, U., Emrich, H.M. (2002). Persisting continuous visual perception disorder in a chronic MDMA ('ecstasy') user. *Aust. N. Z. J. Psychiatry.* 36, 266-267.

Rosenthal, S.H. (1964). Persistent hallucinosis following repeated administration of hallucinogenic drugs. *Am. J. Psychiatry*. 121, 238-244.

Saidel, D.R., and Babineau, R. (1976). Prolonged LSD flashbacks as conversion reactions. *J. Nerv. Ment. Dis*. 163, 352-355

Schatten, H., Eter, N., Mihailovic, N. (2020). „Visual snow” bei „Hallucinogen Persisting Perception Disorder”. *Ophthalmologe*. 117, 1112-1115.

Scott, M.E. (1971). The flashback phenomenon. *Va. Med. Mon. (1918).* 98, 317-320.

Skryabin, V.Y., Vinnikova, M., Nenastieva, A., Alekseyuk, V. (2018). Hallucinogen persisting perception disorder: A literature review and three case reports. *J. Addict. Dis.* 37, 268-278.

Stanciu, C.N., and Penders, T.M. (2016). Hallucinogen persistent perception disorder induced by new psychoactive substituted phenethylamines; A review with illustrative case. *Curr. Psychiatry. Rev.* 12, 221-223.

Subramanian, N., and Doran, M. (2014). Improvement of hallucinogen persisting perception disorder (HPPD) with oral risperidone: Case report. *Ir. J. Psychol. Med*. 31, 47-49.

Sullivan, J.F. (2013). Case report: Hallucinogen persisting perception disorder after exposure to ayahuasca. *J. Neuropsychiatry. Clin. Neurosci*. 25, 31 [conference abstract].

Sunness, J.S. (2004). Persistent afterimages (palinopsia) and photophobia in a patient with a history of LSD use. *Retina*. 24, 805.

Thurlow, H.J., and Girvin, J.P. (1971). Use of anti-epileptic medication in treating "flashbacks" from hallucinogenic drugs. *Can. Med. Assoc. J*. 105, 947-948.

Wentworth-Rohr, I. (1970). Marijuana flashback: A clinical note. *Psychotherapy: Theory, Research & Practice.* 7, 236-237.

Wodarz, N., and Böning, J. (1993). „Ecstasy"-induziertes psychotisches Depersonalisationssyndrom. *Nervenarzt*. 64, 478-480.

Woody, G.E. (1970). Visual disturbances experienced by hallucinogenic drug abusers while driving. *Am. J. Psychiatry*. 127, 683-686.

Young, C.R. (1997). Sertraline treatment of hallucinogen persisting perception disorder. *J. Clin. Psychiatry*. 58, 85.

Zobor, D., Strasser, T., Zobor, G., Schober, F., Messias, A., Strauss, O., et al. (2015). Ophthalmological assessment of cannabis-induced persisting perception disorder: Is there a direct retinal effect? *Doc. Ophthalmol.* 130, 121-130.

Zweben, J.E., Clark, H.W., Smith, D.E. (1994). Traumatic experiences and substance abuse: Mapping the territory. *J. Psychoactive. Drugs*. 26, 327-344.
